# Reoperation and mortality following elective surgery for chronic and recurrent colonic diverticular disease: A nationwide population-based cohort study

**DOI:** 10.1007/s00384-025-04915-9

**Published:** 2025-05-17

**Authors:** Helene R. Dalby, Rune Erichsen, Kaare A. Gotschalck, Katrine J. Emmertsen

**Affiliations:** 1 Department of Surgery, Randers Regional Hospital, Randers, Denmark; 2 Department of Clinical Epidemiology, Aarhus University Hospital & Aarhus University, Aarhus, Denmark; 3 Department of Surgery, Horsens Regional Hospital, Horsens, Denmark; 4 Department of Clinical Medicine, Aarhus University, Aarhus, Denmark

**Keywords:** Chronic diverticular disease, Recurrent diverticular disease, Reoperation, Mortality, Anastomotic leak

## Abstract

**Purpose:**

The ideal treatment for chronic and recurrent colonic diverticular disease (crDD) remains unresolved, partly due to lacking evidence regarding surgical safety. This study evaluated 90-day reoperation and mortality rates following elective surgery for crDD and explored predictors for reoperation and mortality.

**Methods:**

This national cohort study included all patients with crDD undergoing elective colonic resection or stoma formation in Denmark from 1996–2021. Outcomes were the 90-day cumulative incidence proportion (CIP) of reoperation and mortality, and predictors were explored in Cox proportional hazard models.

**Results:**

Among 35,174 patients with crDD, 3,584 (10%) underwent elective surgery. The 90-day reoperation rate was 18.0%; mortality was 1.6%. During the 25-year period, the reoperation rate declined 30%, from 19.5% to 13.8%, and mortality declined 74%, from 2.7% to 0.7%. Among 2,942 patients with colonic resection and no stoma formation, the reoperation rate due to anastomotic leak was 3.0% overall and 0.9% in the most recent years. Mortality was 18 times higher in patients aged ≥ 80 years versus those aged < 60 years (CIP 8.0% versus 0.4%). The reoperation rate was increased in patients with ≥ 4 hospital contacts or ≥ 3 admissions compared to patients with fewer contacts, while mortality was not associated with the number of hospital contacts before surgery.

**Conclusion:**

Elective surgery in crDD was safe with careful patient selection. The risk of reoperation due to anastomotic leak was very low. Patients with most hospital contacts had an increased reoperation rate, supporting consideration for elective surgery early in patients with disabling diverticular disease.

**Supplementary Information:**

The online version contains supplementary material available at 10.1007/s00384-025-04915-9.

## Introduction

After an initial episode of colonic diverticulitis, recurrence is 12–48% if treated without resection [1–5]. The ideal management of recurrences, persisting symptoms, or chronic complications, including stenosis and fistula, remains debated internationally. A central challenge in both clinical practice and research is the lack of an internationally accepted definition of chronic and recurrent diverticular disease (crDD), resulting in variability in terminology and inclusion criteria across studies. In the present study, the term crDD is used to describe patients with recurrent, persisting, or complicated colonic diverticular disease requiring elective surgical intervention.

During recent decades, recommendations have shifted from advising that all patients should have elective colonic resection after their second episode of diverticulitis towards a tailored approach balancing the patients’ symptom burden against the risks accompanying surgery [6–8].

Existing research on the reoperation rate and mortality following elective colonic resection or stoma formation for crDD has limited generalisability due to selected patient cohorts [1, 9–14], small sample sizes [9, 12, 15–26] or settings in healthcare systems with a narrow uptake area and/or selected socio-economic population [14, 21, 27, 28]. Extensive, contemporary studies are needed to assess current practices, inform patient counselling and guide patient selection when considering elective surgery versus conservative treatment for crDD.

The objectives of the current study were to analyse the 90-day reoperation and mortality rates following elective colonic resection or stoma formation for crDD. Furthermore, predictors for reoperation and death were explored to identify which patients could safely undergo elective surgery.

## Methods

### Setting

This cohort study was based on the national Danish population from 1996 to 2021. The Danish national healthcare system provides unrestricted universal tax-supported healthcare to all legal residents of Denmark (around 5.8 million) [29].

The study was approved by the Danish Data Protection Agency (Central Denmark Region) (record no. 2016–051-000001, 2624). The study was based on anonymised registry data and, therefore, was exempt from informed consent following the General Data Protection Regulations. The study was reported following the *Strengthening the Reporting of Observational Studies in Epidemiology* (STROBE) guideline (Supplementary Table 1) [30].

### Data sources

Data sources included the Danish Civil Registration System (CRS) and the Danish National Registry of Patients (DNRP), linked via the unique personal civil registration (CPR) number assigned to all Danish residents at birth or upon immigration. The CRS includes data on sex, date of birth, death, and emigration with high validity and completeness [31, 32]. The DNRP contains data on all hospital contacts and surgical procedures from all Danish hospitals. Inpatient contacts are classified as elective or emergency. Diagnoses have been coded using the International Classification of Diseases, Tenth Revision (ICD-10) since 1994, and surgeries have been coded using the NOMESCO Classification of Surgical Procedures since 1996. Codes used in the study are listed in Supplementary Table 2.

### Study cohort

The study cohort included all patients with crDD undergoing elective colonic resection or stoma formation due to crDD. Patients entered the cohort on the date of their first relevant surgery following crDD diagnosis (index date). Patients were defined as having crDD when registered with two relevant hospital contacts within 5 years. Relevant hospital contacts were inpatient or outpatient contacts with a primary diagnosis of DD or complications to DD, including colonic stenosis or fistula with a preceding diagnosis of DD. To avoid the inclusion of asymptomatic diverticulosis, outpatient visits with endoscopy were excluded as relevant contacts.

The DNPR was used to identify the study cohort. The positive predictive value of DD in the DNPR is 98% [33]. Data in the DNPR were available from 1977 to 2021, but only patients with crDD diagnosis after January 1 st, 1996, were included due to the implementation of the NOMESCO classification of surgical procedures in 1996. Patients who had undergone any colonic resection or stoma formation before crDD diagnosis were excluded. Patients were included until October 1 st 2021, to ensure 90 days follow-up.

### Reoperation and mortality

Reoperations within 90 days after elective colonic resection or stoma formation for crDD were identified in the DNPR from procedure codes. Reoperations were categorised into eight reoperation types. Mortality dates were extracted from the CRS.

### Potential predictors

Information on potential predictors for reoperation and mortality was retrieved from the DNPR. Comorbidity was classified according to the Charlson Comorbidity Index (CCI) [34]. Disease severity was classified as complicated if any contact before or at the surgery involved an abscess, perforation, stenosis, or fistula. The disease duration was defined as days from the first relevant hospital contact until surgery, with relevant contacts defined as above. The contact count was defined as the total number of relevant hospital contacts before surgery, and the admission count was the number of admissions due to DD before surgery. The year of surgery was categorised into five intervals. The surgical approach, procedure, and type of colonic resection were extracted from the procedure codes. The surgical approach was categorised as minimally invasive (MIS) if performed by laparoscopy or robot-assisted and otherwise as open. The surgical procedure was classified as colonic resection with or without stoma formation or stoma formation alone.

### Statistical analysis

Patients with crDD were followed from the index date until 90 days postoperatively. Descriptive statistics were compiled for all patients at the index date. Quantitative data were estimated as median with interquartile range (IQR) and categorical data as absolute numbers and percentages.

The absolute risk of reoperation was estimated as the cumulative incidence proportion (CIP) at 90 days, treating death as a competing event and migration as a censoring event. CIPs were calculated for overall reoperations and different reoperation types. CIP of reoperation due to anastomotic leak was only estimated for patients with colonic resection and no stoma formation at index surgery. The mortality was calculated as the 90-day CIP of death, treating migration as a censoring event. CIP for reoperation and death were stratified according to the possible predictors and reported with 95% confidence intervals (CIs). CIP curves were visualised using the Aalen-Johansen estimator.

The association between potential predictors and incidence of reoperation and death were evaluated using Cox proportional hazard models. The predictors included sex, age, CCI score, disease severity, year of surgery, disease duration, surgical approach, and surgical procedure. Due to the presumed multicollinearity between the disease duration, contact count, and admission count, contact count and admission count were analysed separately, adjusting for the remaining variables, excluding disease duration. Cause-specific hazard ratios (HRs) with 95% CIs were reported. Adjusted HRs were visualised in forest plots. HR for reoperation was calculated, treating death and migration as censoring events. To explore the associations between age and mortality without imposing arbitrary cut-offs, age was modelled as a continuous variable using restricted cubic splines with four knots, placed at percentiles defined by Harrell’s default method [35].

### Sensitivity analyses

Three sensitivity analyses were conducted to examine the robustness of the crDD definition. Firstly, the cohort was restricted to patients with at least three relevant hospital contacts before surgery. Secondly, it was limited to only include patients with at least *two admissions* due to DD before surgery. Thirdly, it was restricted to only include patients with complicated disease. CIP and HRs for surgery were estimated as described previously.

### Stoma formations and changes over the study period

The proportion of patients with a stoma by postoperative day 90 was calculated, excluding stoma reversals. Changes in patient characteristics and procedures over the study period were described using descriptive statistics. The CIP of reoperation due to anastomotic leak for all patients with colonic resection and no stoma formation at the index surgery was calculated as described above and stratified according to the year of surgery.

Statistical analyses were performed using RStudio, version 2023.12.1 (Posit PBC). Data were analysed from January to September 2024.

## Results

Among 35,174 patients diagnosed with crDD between 1996 and 2021, 3,584 (10%) patients underwent elective colonic resection or stoma formation. The median age was 62 (IQR: 53–71) and females constituted 60%. Most patients undergoing elective surgery had no or few comorbidities (Table 1). The median disease duration before the index date was 240 (IQR: 69–1,233) days, and surgery was performed on the index date in 735 (21%) patients. Most patients had a colonic resection without stoma formation, while stoma formation was performed in 637 (18%) of the procedures. Patients with stoma formation at the index surgery had a median age of 67 (IQR: 58–76), and 57% had no comorbidities, while those with no stoma formation had a median age of 61 (IQR: 53–70), and 59% had no comorbidities. Stoma formation with no colonic resection was performed in 2% of patients aged < 75, 5% of patients aged 75–79 years, 8% of patients aged 80–84 years, and 19% of patients aged 85 +. Overall, 2,196 (61%) of procedures were performed by an open approach, but while 77% of patients with stoma formation underwent open surgery, only 58% of those with no stoma formation underwent open surgery.

**Table 1:** Characteristics of patients with chronic and recurrent diverticular disease at the time of elective surgery and the procedures performed (number (%)). a) Complicated if any contact before or at the surgery was with abscess/perforation, stenosis, or fistula b) 2016-2021 includes 5 years and 9 months, whereas the other 4 groups include 5 years only

		Number of patients (*n* = 3,584)
**Sex**	Female	2,155 (60)
	Male	1,429 (40)
**Age group**	< 60	1,482 (41)
	60–69	1,065 (30)
	70–79	812 (23)
	80 +	225 (6)
**CCI score**	0	2,089 (58)
	1–2	1,187 (33)
	3 +	308 (9)
**Severity**	Uncomplicated	2,067 (58)
	Complicated ^a^	1,517 (42)
*Subgroups of complicated cDD*	*Abscess or perforation*	*695 (19)*
	*Fistula*	*682 (19)*
	*Stenosis*	*338 (9)*
**Disease duration** (time from first relevant hospital contact to surgery)	0–30 days	430 (12)
	30–364 days	1,603 (45)
	1 + years	1,551 (43)
**Contact count** (number of relevant hospital contacts before surgery)	1	666 (19)
	2	720 (20)
	3	611 (17)
	4 +	1,587 (44)
**Admission count** (number of admissions due to diverticular disease before surgery)	0	938 (26)
	1	1,188 (33)
	2	699 (20)
	3 +	759 (21)
**Year of surgery**	1996–2000	476 (13)
	2001–2005	623 (17)
	2006–2010	705 (20)
	2011–2015	743 (21)
	2016–2021 ^b^	1,037 (29)
**Approach**	Minimally invasive	1,388 (39)
	Open*	2,196 (61)
	* Converted procedures	*460 (13)*
**Surgical procedure**	Resection, no stoma	2,947 (82)
	Resection with stoma	531 (15)
	Stoma, no resection	106 (3)
**Type of colonic resection** (n = 3,478)	Left hemicolon, sigmoid colon, rectum	3,405 (98)
	Other colonic resection	73 (2)

The median length of stay after the procedure was 6 (IQR: 4–10) days.

### Reoperation

**Fig. 1:**
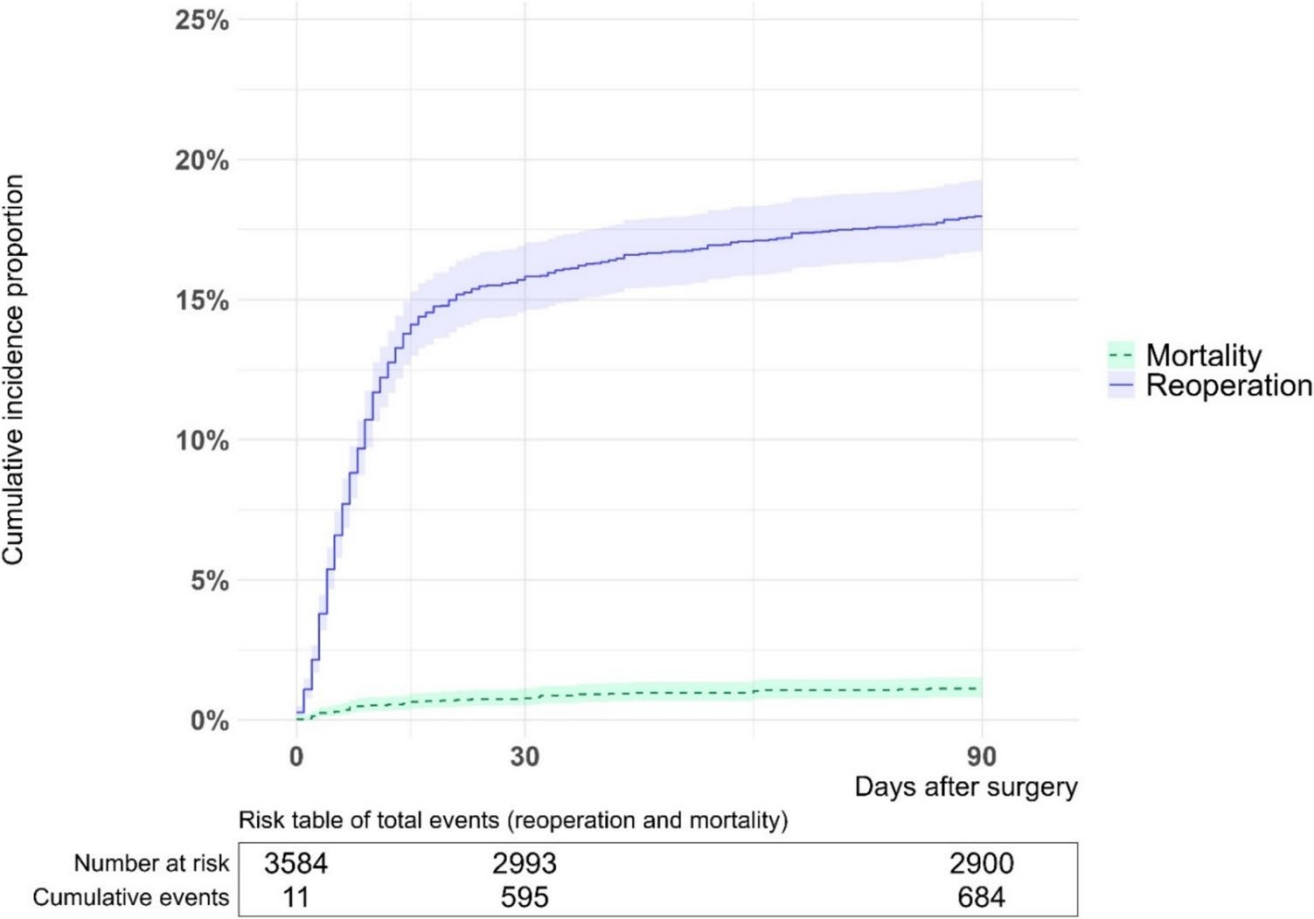
Cumulative incidence proportion of reoperation and mortality following elective colonic resection or stoma formation for chronic and recurrent diverticular disease

The overall 90-day CIP of reoperation was 18% (Table 2 and Fig. 1). The CIP of reoperation was 19–22% during the years 1996–2015, whereafter it declined to 14% in 2016–2021. The CIP of reoperation was 21% among males and 16% among females. CIP of reoperation following open surgery was 21%, whereas CIP following MIS was 14%. Similarly, patients with no stoma formation at index surgery had a CIP of reoperation at 17%, while patients with stoma formation and no colonic resection had a CIP of reoperation at 24%. CIP of reoperation was approximately the same within the other stratifications (Table 2).

**Table 2:** Cumulative incidence proportion (CIP) of reoperation and death within 90 days of surgery for chronic and recurrent diverticular disease. Numbers are CIP in % (95% confidence intervals in %). a) Complicated if any contact before or at the surgery was with abscess/perforation, stenosis, or fistula b) 2016-2021 includes 5 years and 9 months, whereas the other 4 groups include 5 years only

		Reoperation	Mortality
**Overall**		18.0 (16.7–19.2)	1.6 (1.2–2.0)
**Sex**	Female	16.1 (14.5–17.6)	1.5 (1.1–2.1)
	Male	20.9 (18.8–23.0)	1.6 (1.1–2.4)
**Age group**	< 60	18.2 (16.2–20.2)	0.4 (0.2–0.9)
	60–69	17.3 (15.1–19.6)	1.2 (0.7–2.0)
	70–79	18.5 (15.9–21.2)	2.3 (1.5–3.6)
	80 +	18.2 (13.5–23.5)	8.0 (4.9–12.0)
**CCI score**	0	18 0 (16.4–19.7)	1.6 (1.1–2.2)
	1–2	17.8 (15.7–20.0)	1.6 (1.0–2.4)
	3 +	18.5 (14.4–23.0)	1.0 (0.3–2.7)
**Severity**	Uncomplicated	16.9 (15.4–18.6)	1.2 (0.8–1.7)
	Complicated ^a^	19.4 (17.4–21.4)	2.1 (1.5–2.9)
**Disease duration** (time from first relevant hospital contact to surgery)	0–30 days	18.4 (14.9–22.2)	3.3 (1.9–5.3)
	30–364 days	18.7 (16.8–20.7)	1.3 (0.8–2.0)
	1 + years	17.1 (15.3–19.0)	1.4 (0.9–2.0)
**Contact count** (number of relevant hospital contacts before surgery)	1	17.1 (14.4–20.1)	2.6 (1.5–4.0)
	2	18.7 (16.0–21.7)	1.5 (0.8–2.0)
	3	17.5 (14.6–20.6)	1.6 (0.8–2.9)
	4 +	18.1 (16.3–20.1)	1.1 (0.7–1.8)
**Admission count** (number of admissions due to diverticular disease before surgery)	0	18.3 (15.9–20.9)	1.5 (0.9–2.4)
	1	17.8 (15.6–20.0)	1.6 (1.1–2.6)
	2	14.7 (12.2–17.5)	1.3 (0.6–2.4)
	3 +	20.8 (18.0–23.8)	1.6 (0.9–2.7)
**Year of surgery**	1996–2000	19.5 (16.1–23.2)	2.7 (1.5–4.5)
	2001–2005	21.5 (18.4–24.8)	1.8 (0.9–3.0)
	2006–2010	18.9 (16.1–21.8)	1.3 (0.6–2.3)
	2011–2015	19.0 (16.2–21.9)	2.2 (1.3–3.4)
	2016–2021 ^b^	13.8 (11.8–16.0)	0.7 (0.3–1.3)
**Approach**	Minimally invasive	13.5 (11.7–15.3)	0.6 (0.3–1.1)
	Open	20.8 (19.1–22.5)	2.2 (1.6–2.9)
**Surgical procedure**	Resection, no stoma	17.1 (11.7–15.3)	1.0 (0.6–1.4)
	Resection with stoma	21.5 (18.1–25.1)	3.8 (2.4–5.6)
	Stoma, no resection	23.6 (16.0–32.0)	7.5 (3.5–13.6)

The CIP of different reoperations are listed in Table 3. The most frequent reason for reoperation was wound complications, including wound rupture, infection, or revision, with a CIP of 6%. CIP of reoperation due to anastomotic leak for patients with colonic resection and no stoma formation was 3%. CIP of reoperation due to anastomotic leak was 3.4% (CI: 1.9–5.4) in 1996–2000, peaked at 6.5% (CI: 4.7–8.6) in 2006–2010, and declined to 0.9% (CI:0.4–4.9) in 2016–2021. According to the surgical approach, further stratification of the surgeries in 2016–2021 revealed a CIP of reoperation due to anastomotic leak at 0.8% (0.2–2.2) following open surgery and 0.2% (0.0–0.8) following MIS.

**Table 3:** Cumulative incidence proportion (CIP) of types of reoperations within 90 days of surgery for chronic and recurrent diverticular disease. a) Other reoperations include lavage, drain, diagnostic procedures, endoscopic therapy of gastrointestinal bleeding, or suture of gastroduodenal perforation

	At risk	*n*	Postoperative day *median (IQR)*	90-day CIP *% (95% CI)*
**Mortality**	3,584	65	16 (6–32)	1.6 (1.2–2.0)
**Overall reoperations**	3,584	644	9 (4–15)	18.0 (16.7–19.2)
Anastomotic leak	2,947	106	6 (4–9)	3.0 (2.4–3.6)
Stoma formation not due to anastomotic leak	2,947	87	6 (4–10)	2.4 (2.0–3.0)
Stoma revision	637	14	10 (5–22)	0.4 (0.2–0.6)
Surgical site infection, deep	3,584	44	14 (9–36)	1.2 (0.9–1.6)
Bleeding, deep or superficial	3,584	21	1 (0–2)	0.6 (0.4–0.9)
Reconstruction in urinary tract	3,584	8	10 (7–18)	0.2 (0.1–0.4)
Wound complications (dehiscence, infection, revision)	3,584	222	12 (8–18)	6.2 (5.4–7.0)
Other reoperation ^a^	3,584	142	8 (4–29)	4.0 (3.4–4.6)

Overall, the median day of reoperation was postoperative day 9 (IQR: 4–15), and the median day of reoperation due to anastomotic leak was day 6 (IQR: 4–9). The timing of reoperation types is presented in Table 3.

### Mortality

The 90-day CIP of mortality following elective surgery for crDD was 1.6% (Table 2 and Fig. 1), and the median postoperative day of death was day 16 (IQR: 6–32) (Table 3).

The CIP of mortality was exceptionally high in patients aged ≥ 80 years at 8%, while patients < 60 years had much lower mortality at 0.4%. The CIP of mortality was 2.7% during 1996–2000 and declined to 0.7% in 2016–2021. Stratifying CIP during the years 2016–2021 by age groups revealed mortality of 0.5% (CI: 0.1–1.6) for patients < 60 years of age, 0.9 (CI: 0.3–2.6) for patients aged 60–69 years, 0% for patients aged 70–79 years, and 3.3% (CI: 0.6–10.2%) for those aged ≥ 80 years.

Additionally, the mortality was high at 7.5% in patients with stoma formation only, while those with colonic resection without stoma formation had a mortality of 1%.

### Potential predictors for reoperation and death

#### Reoperation

**Fig. 2:**
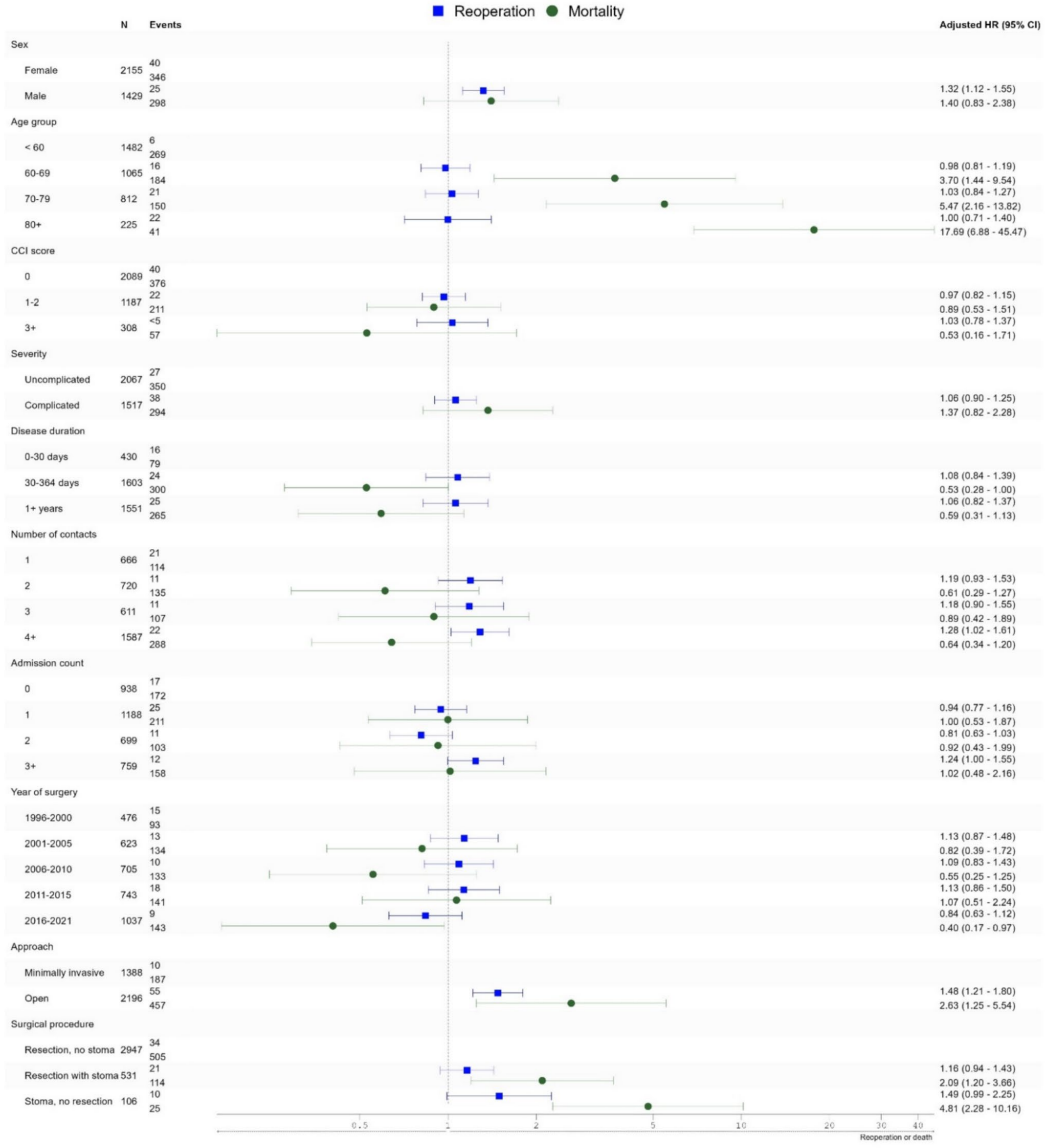
Hazard ratio (HR) for reoperation and mortality following elective colonic resection or stoma formation for chronic and recurrent diverticular disease. Numbers are adjusted HRs. Estimates for sex, age group, CCI score, severity, disease duration, year of surgery, approach, and surgical procedure are mutually adjusted. The number of contacts and admission count were analysed separately and adjusted for the remaining variables, excluding disease duration

Predictors for reoperation included male sex and surgery by open approach. Furthermore, the reoperation rate was higher in patients with more than four hospital contacts and in those with three or more admissions (Fig. 2). Minimal differences were observed between crude and adjusted HRs (Supplementary Table 3).

#### Mortality

Increasing age was the strongest predictor for mortality (Fig. 2). When modelled as a continuous variable using restricted cubic splines, age showed a non-linear association with 90-day mortality. The hazard of death increased modestly from age 60 to 75, followed by a steeper rise beyond age 75. This pattern remained consistent after adjustment. Neither contact nor admission count predicted mortality. Stoma formation at index surgery was strongly associated with increased mortality, even after adjusting for age (Supplementary Table 3). An open surgical approach was also associated with higher mortality.

Further analysis of the association between potential predictors and a composite outcome of reoperation and death revealed results similar to those for reoperation alone.

### Sensitivity analyses

The two sensitivity analyses restricting the cohort based on the number of contacts provided consistent estimates of the CIPs of reoperation and mortality as well as HRs of the possible predictors. In 2,918 patients with at least *three* relevant hospital contacts before surgery, the CIP of reoperation was 18.2% (CI: 16.8–19.6), and the mortality rate was 1.3%. In 1,458 patients with at least *two admissions* before surgery, the CIP of reoperation was 17.9% (CI: 16.0–19.9), while mortality was 1.4%. The analysis of the 1,517 patients with complicated disease additionally revealed consistent estimates of the HRs of the possible predictors for reoperation and mortality.

### Stoma formations

Among 2,947 patients with no stoma formation at the index surgery, 141 had stoma formation during reoperation. This adds up to 778 (21.7%) stoma formations. Within the 90-day follow-up, 42 (5.4%) patients had their stomas closed. The median postoperative day of stoma closure was day 61 (IQR: 31.8–79.2). Of the 3,519 patients alive 90 days after elective surgery, 736 (20.9%) had a stoma at postoperative day 90.

### Changes during the study period

**Fig. 3:**
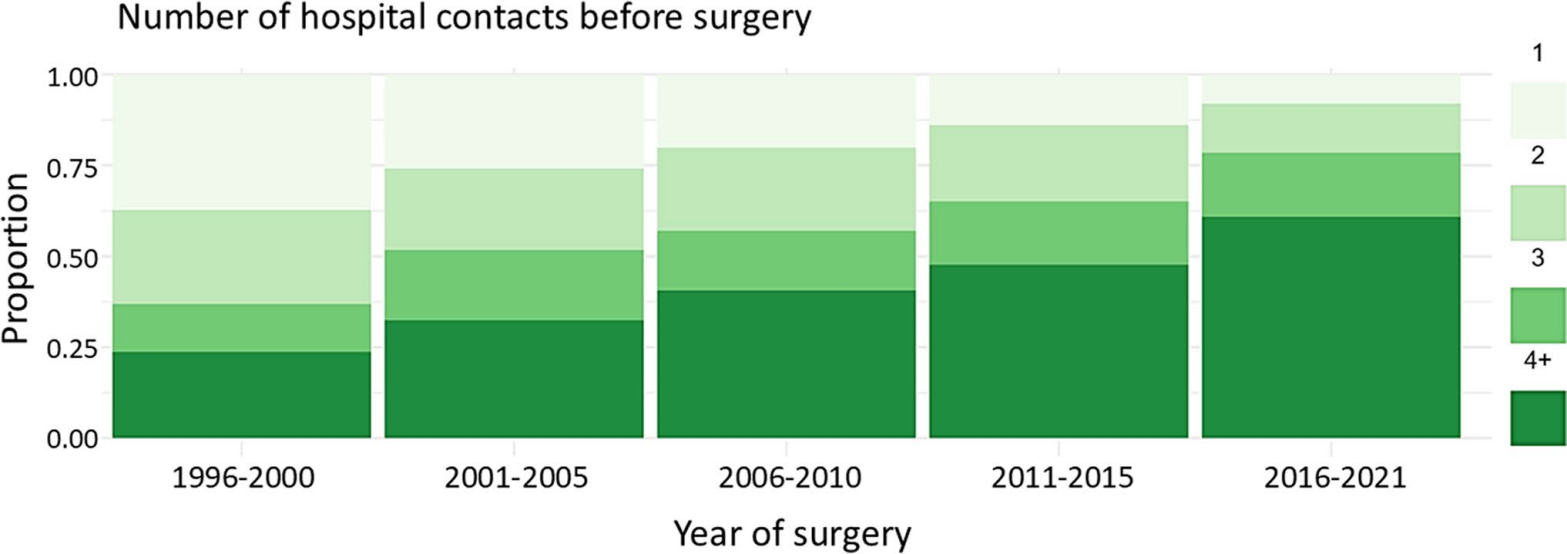
Proportion of patients undergoing elective colonic resection or stoma formation for chronic and recurrent diverticular disease according to number of hospital contacts before surgery, stratified by year of surgery

Over the study period, the median contact count before surgery increased from 2 (IQR: 1–2.8) contacts in 1996 to 4 (IQR: 3–6) contacts in 2021 (Fig. 3). Accordingly, the median disease duration prior to surgery increased from 99 (IQR: 38–382) days in 1996–2000 to a median of 521 (IQR: 108–1,731) days in 2016–2021.

Additionally, the surgical approach changed over the study period. The proportion of MIS was 1% in 1996–2000. There was a remarkable rise in MIS from 2004 (10%) to 2007 (40%), and from 2015 onwards, MIS proportion was above 60%. The proportion of stoma formations was constant, around 18%, over the study period.

## Discussion

This study represents the first large-scale analysis of a non-selected national cohort within an unrestricted, tax-funded healthcare system investigating reoperation and mortality following elective colonic resection or stoma formation for crDD. It provides valuable insights into current surgical practices and outcomes. Our findings show a remarkable improvement in reoperation rates and mortality over the past decades and a very low risk of reoperation due to anastomotic leak. In 2016–2021, approximately one in seven patients required reoperation and only one in 100 required reoperations due to anastomotic leak. Postoperative mortality was one in 143.

Although 90-day mortality declined over the study period, increasing age remained the strongest predictor of mortality, underscoring the need for caution in older patients. The association between age and mortality was non-linear, with a steep rise in age groups above 75 years. This supports a cautious approach to elective surgery in this group, especially in the presence of additional risk factors. However, the impact of age may be overestimated due to unmeasured confounders not captured in the available data, including frailty, cognitive status, and lifestyle factors, and should not be interpreted as a sole indicator of surgical complications. In contrast, comorbidity did not predict reoperation or mortality rates. Reoperation rates were not associated with age or comorbidity, which may be due to patient selection – for example, frail patients may have been more likely to undergo less invasive procedures, such as stoma formation with no colonic resection. Additionally, a reluctance to perform reoperations in the most frail patients cannot be excluded.

Our results show that postoperative mortality and reoperation rates for crDD have declined by more than 70% and 30%, respectively, over recent decades. This is likely attributable to improvements in surgical quality, patient selection, and perioperative care. Correspondingly, the proportion of MIS increased from 0% to over 60% during the study period.

A very low rate of anastomotic leak was observed among patients who underwent surgery between 2016 and 2021, with an even lower rate in those undergoing MIS.

Although the present study did not include data on the indications for stoma formation or the surgical approach used, the findings highlight the safety of current practices. Patients selected for anastomosis rarely required reoperation due to anastomotic leak. Most reoperations were performed for wound-related complications, such as dehiscence, infection, or revision. While more detailed information on the specific indications for reoperation was unavailable, careful postoperative wound management remains essential in this patient population.

CrDD encompasses a heterogeneous group of patients, ranging from those managed in primary care to those requiring recurrent hospital admissions. The heterogeneity underscores the challenges in defining and categorising crDD, particularly in epidemiological studies, where case identification relies on administrative coding rather than clinical criteria. As no universally accepted definition of crDD exists, this study employed a register-based definition informed by prior clinical studies to identify patients with ongoing or recurrent symptoms [1, 10, 36, 37]. While administrative data offer large patient cohorts, this approach may introduce misclassification bias, particularly in distinguishing between patients with chronic debilitating symptoms and those who experience only a few mild episodes that resolve quickly and are otherwise asymptomatic. Two sensitivity analyses were conducted to evaluate the robustness of the applied definition, with consistent findings observed for reoperation, mortality, and risk predictors. Specifically, these analyses applied narrower diagnostic criteria to strengthen case ascertainment. The consistency of results across these approaches suggests that the observed associations are unlikely to be driven by misclassification. This reinforces the validity of the primary findings and highlights the reliability of administrative data in capturing clinically relevant outcomes in crDD.

Population-based estimates of reoperation and mortality following surgery for crDD remain limited. Prior studies focusing on elective colonic resections report reoperation rates of 4–12%, mortality rates of 0–1%, and stoma rates of 5–18% [9, 11–14], which are slightly lower than those observed in our study. This discrepancy may reflect our inclusion of patients undergoing stoma formation without colonic resection, who are likely to present with more severe disease or comorbidities. In line with this observation, higher reoperation and mortality rates were noted in patients requiring stoma formation than those undergoing resections alone. Nonetheless, we observed a decreasing incidence of reoperation and mortality over the study period, with the rates from the last decade comparable to prior studies.

Clinical trials have investigated surgical outcomes in younger and healthier patients with crDD, reporting reoperation rates of 10–23%, no mortality, and a stoma rate of 2–11% after 90 days [1, 10]. However, the limited generalisability of these findings to older or comorbid patients highlights the relevance of our study, which encompasses a broader and more representative patient population.

Our study highlights that surgical approach and procedure are essential for postoperative reoperation and mortality rate, although confounding by indication may partly explain our findings. We could not assess details of the surgical procedures performed, including variations in surgical expertise. Still, it is likely that patients necessitating an open approach are more challenging than patients successfully operated by MIS and that patients requiring stoma formation are frailer and/or more contaminated than patients not requiring a stoma formation. Accordingly, previous cohort studies have found open surgical approach as well as patient factors, including comorbidities, obesity, and smoking, to be risk factors for complications following elective resections for crDD [11, 13, 14]. Although our study did not include data on obesity or smoking status, we found no association between the CCI score and reoperation rate or mortality. Several factors may explain this finding. It has previously been found that older and comorbid patients with crDD had a very low incidence of elective surgery compared to younger or healthier patients [38]. Accordingly, the proportion of patients with comorbidities in our cohort was relatively low (less than 10%), leading to a cohort with relatively favourable baseline health. This may have limited our ability to detect significant differences. Additionally, the CCI may not fully capture comorbidities relevant to postoperative outcomes in crDD, such as obesity or nutritional status. Lastly, advances in perioperative care, including optimised anaesthetic and postoperative management protocols, may have mitigated the impact of comorbidities on surgical outcomes.

Over the study period, an increase in hospital contacts prior to surgery was observed, likely reflecting a shift towards more conservative management in accordance with evolving guidelines. Importantly, no association was found between the number of hospital contacts and mortality, suggesting the safety of current practices. Nonetheless, HR for reoperation was higher in patients with ≥ 4 hospital contacts or ≥ 3 admissions compared to patients with fewer contacts, highlighting the need for continuous consideration of the indications for elective surgery with increasing hospital contact.

Surgery within 30 days of first contact seemed associated with higher mortality than longer disease duration. Although all included procedures were coded as elective, a shorter interval from diagnosis to surgery may reflect unmeasured clinical urgency, treatment failure, or severe symptomatology necessitating early intervention. This raises the possibility that certain procedures, while coded as elective, may in practice have been performed in a semi-urgent setting. In defining the study cohort, patients who underwent surgery during the same admission as their initial emergency presentation were excluded, as these cases are more likely to reflect acute disease rather than the chronic or recurrent symptoms that characterise crDD. However, this exclusion may have omitted a subgroup of patients with chronic or recurrent disease who progressed rapidly and underwent surgery shortly after emergency admission. While the intention was to capture a well-defined elective population with chronic disease, this approach may underestimate the spectrum of disease severity and the outcomes of semi-acute surgery. This limitation should be considered when interpreting the association between the timing of surgery and postoperative outcomes.

### Strengths and limitations

The strengths of the present study include a nationwide cohort and the investigation of real-world data, including older and comorbid patients, who are often not eligible for inclusion in clinical trials. The study thereby evaluates the outcomes after surgery in patients with crDD in Denmark over the past 25 years. The sample size was large, and all data were available for the entire cohort.

The limitations of this study include those associated with the use of administrative data. The lack of access to individual patient charts precluded validation of coding, potentially causing misclassification, and retrieval of more granular clinical data, such as smoking status or body mass index, potentially leading to unmeasured confounding. Additionally, postoperative complications that do not lead to reoperation have not been analysed but may impact patients. While a chart review could have strengthened the study by confirming diagnostic accuracy, surgical indications, and postoperative events, the DNPR has demonstrated high validity for surgical procedures and diagnoses [39], which supports the reliability of the findings. Future studies could benefit from linkage with the Danish Pathology Registry to enable histological validation and more granular classification of disease severity.

Indications for surgery, operative techniques, and perioperative management likely evolved over the 25-year study period. Although coding practices were consistent and stratification was applied in the analyses to mitigate these temporal effects, some heterogeneity may not have been fully accounted for. Additionally, the register-based definition of crDD excludes chronically affected patients without hospital contact.

## Conclusions

This nationwide cohort study provides contemporary, population-based estimates of short-term reoperation and mortality following elective surgery for crDD. The findings highlight improvements in surgical outcomes over time while underscoring the need for careful patient selection, particularly among older individuals. Accordingly, elective surgery was safe in younger and comorbid patients. The number of hospital contacts before surgery was not associated with post-operative mortality, supporting current guidelines recommending elective surgery based on individual assessment rather than solely the number of hospital contacts. The study addresses a gap in the literature by including non-selected patients undergoing elective surgery, offering insights applicable to broader clinical practice. While this study focused on surgical safety, future research should incorporate patient-reported outcomes to evaluate the wider effectiveness of crDD management strategies.

## Supplementary Information

Below is the link to the electronic supplementary material.

Supplementary file1 (DOCX 66 KB)

## Data Availability

No datasets were generated or analysed during the current study.
