# Supplementary material for: Reoperation and mortality following elective surgery for chronic and recurrent colonic diverticular disease: A nationwide population-based cohort study
Source: Int J Colorectal Dis. 2025 May 17;40(1):123. doi: 10.1007/s00384-025-04915-9 (PMC12085347; doi:10.1007/s00384-025-04915-9)
Supplement: Supplementary file 1 — Supplementary file1 (DOCX 66 KB) [file 384_2025_4915_MOESM1_ESM.docx]

## **Supplementary table 1**: STROBE Checklist of items that should be included in *cohort studies*

|  | Item | Recommendation | Page |
| --- | --- | --- | --- |
| **Title and abstract** | 1 | (*a*) Indicate the study design with a commonly used term in the title or the abstract | Title |
|  |  | (*b*) Provide in the abstract an informative and balanced summary of what was done and what was found | 2 |
| Introduction | | |  |
| Background/rationale | 2 | Explain the scientific background and rationale for the investigation being reported | 3 |
| Objectives | 3 | State specific objectives, including any prespecified hypotheses |  |
| Methods | | |  |
| Study design | 4 | Present key elements of study design early in the paper | 3 |
| Setting | 5 | Describe the setting, locations, and relevant dates, including periods of recruitment, exposure, follow-up, and data collection | 3-5 |
| Participants | 6 | (*a*) Give the eligibility criteria, and the sources and methods of selection of participants. Describe methods of follow-up | 4 |
|  |  | (*b*) For matched studies, give matching criteria and number of exposed and unexposed | NA |
| Variables | 7 | Clearly define all outcomes, exposures, predictors, potential confounders, and effect modifiers. Give diagnostic criteria, if applicable | 4-5 |
| Data sources/ measurement | 8 | For each variable of interest, give sources of data and details of methods of assessment (measurement). Describe comparability of assessment methods if there is more than one group | 5  S-Table 2 |
| Bias | 9 | Describe any efforts to address potential sources of bias | 5-6 |
| Study size | 10 | Explain how the study size was arrived at | 4 |
| Quantitative variables | 11 | Explain how quantitative variables were handled in the analyses. If applicable, describe which groupings were chosen and why | 5-6 |
| Statistical methods | 12 | (*a*) Describe all statistical methods, including those used to control for confounding | 5-6 |
|  |  | (*b*) Describe any methods used to examine subgroups and interactions |  |
|  |  | (*c*) Explain how missing data were addressed |  |
|  |  | (*d*) If applicable, explain how loss to follow-up was addressed |  |
|  |  | (*e*) Describe any sensitivity analyses |  |
| Results | | |  |
| Participants | 13 | (a) Report numbers of individuals at each stage of study—eg numbers potentially eligible, examined for eligibility, confirmed eligible, included in the study, completing follow-up, and analysed | 6 |
|  |  | (b) Give reasons for non-participation at each stage |  |
|  |  | (c) Consider use of a flow diagram |  |
| Descriptive data | 14 | (a) Give characteristics of study participants (eg demographic, clinical, social) and information on exposures and potential confounders | 6  Table 1 |
|  |  | (b) Indicate number of participants with missing data for each variable of interest | NA |
|  |  | (c) Summarise follow-up time (eg, average and total amount) | 6 |
| Outcome data | 15 | Report numbers of outcome events or summary measures over time | 9, Table 2 |
| Main results | 16 | (*a*) Give unadjusted estimates and, if applicable, confounder-adjusted estimates and their precision (eg, 95% confidence interval). Make clear which confounders were adjusted for and why they were included | 7-8  Figure 2  S-Table 3 |
|  |  | (*b*) Report category boundaries when continuous variables were categorized |  |
|  |  | (*c*) If relevant, consider translating estimates of relative risk into absolute risk for a meaningful time period |  |
| Other analyses | 17 | Report other analyses done—eg analyses of subgroups and interactions, and sensitivity analyses | 7-8  Table 3 |
| Discussion | | |  |
| Key results | 18 | Summarise key results with reference to study objectives | 9 |
| Limitations | 19 | Discuss limitations of the study, taking into account sources of potential bias or imprecision. Discuss both direction and magnitude of any potential bias | 11 |
| Interpretation | 20 | Give a cautious overall interpretation of results considering objectives, limitations, multiplicity of analyses, results from similar studies, and other relevant evidence | 9-11 |
| Generalisability | 21 | Discuss the generalisability (external validity) of the study results | 9-11 |
| Other information | | |  |
| Funding | 22 | Give the source of funding and the role of the funders for the present study and, if applicable, for the original study on which the present article is based | Title page |

## **Supplementary table 2**: Diagnosis and procedure codes used in the study

| **Diagnosis** | | ICD-8 (before 1994) | ICD-10 (from 1994 onwards) |
| --- | --- | --- | --- |
| **Diverticular disease and procedures** | | | |
| Diverticulosis | | 5621x | K572-9 |
| Diverticular disease | Uncomplicated | 56210, 56211, 56218, 56219 | K573, K575, K579 |
|  | Complicated | 56212, stenosis, fistula | K272, K574, K578, stenosis, fistula |
| Colonic stenosis | | 56091 | K566, K566A + G, K624B + E |
| Fistula (colovesical, colovaginal, colointestinal) | | 56910, 59601, 59905, 59906, 62989 | K632B, K632I, K632J, N321, N321A, N322, N823, N823A, N824 |
| **Comorbidities for Charlson Comorbidity Index score** | | | |
| Myocardial infarction | | 410 | I21, I22, I23 |
| Congestive heart failure | | 42709-11, 42719, 42899, 78249 | I50, I110, I130, I132 |
| Peripheral vascular disease | | 440, 441, 442, 443, 444, 445 | I70, I71, I72, I73, I74, I77 |
| Cerebrovascular disease | | 430-438 | I60-69, G45, G46 |
| Dementia | | 29009-20919, 29309 | F00-03, F051, G30 |
| Chronic pulmonary disease | | 490-493, 515-518 | J40-47, J60-67, J684, J701, J703, J841, J920, J961, J982, J983 |
| Connective tissue disease | | 712, 716, 734, 446, 13599 | M05-06, M08, M09, M30, M31, M32, M33, M34, M35, M36, D86 |
| Ulcer disease | | 53091, 53098, 531-534 | K221, K25-K28 |
| Mild liver disease | | 571, 57301, 57304 | B18, K700-03, K709, K71, K73-74, K760 |
| Diabetes type 1 and type 2 | | 24900, 24906, 24907, 24909, 25000, 25006, 25007, 25009 | E100-101, E109, E110-111, E119 |
| Diabetes with end organ damage | | 24901-05, 24908, 25001-05, -08 | E102-108, E112-118 |
| Hemiplegia | | 344 | G81, G82 |
| Moderate to severe renal disease | | 403, 404, 580-84, 59009, 59319, 75310-19, 792 | I12-13, N00-N05, N07, N11, N14, N17-N19, Q61 |
| Any tumour | | 140-194 | C00-C75 |
| Leukemia | | 204-207 | C91-C95 |
| Lymphoma | | 200-203, 27559 | C81-C85, C88, C90, C96 |
| Moderate to severe liver disease | | 07000, 07002, 07004, 07006, 07008, 57300, 45600-45609 | B150, B160, B162, B190, K704, K72 K766, I85 |
| Metastatic solid tumour | | 195-198, 199 | C76-C80 |
| AIDS | | 07983 | B21-B24 |
| **Procedure** | | **Surgeries before 1996** | **NOMESCO (from 1996 onwards)** |
| Colonic resection | | 44920-21, 44960-61, 44980-81, 45020-21, 45060-61, 45080-81, 45700, 45720, 45740, 45840-41, 46400, 46440, 46450, 46530, 46490 | JFB2-9, JGB, JFH |
| Stoma formation | | 45180, 45200, 45201, 45210, 45240, 47000, 47100, 47110 | JFF |
| Endoscopies | | 91000, 91010, 91020, 91070, 91080, 92260, 92280, 92300, 92340, 92360, 92490, 93160, 93170, 93210 | UJD, UJF, JFA, JCA, JDA, JDB10 |
| **Outcomes** | | | |
| Anastomotic leakage | | *NA* | JWF |
| Surgical site infection, superficial | |  | JWA, JWB, QBA, QBB, JAA00, bnpa92, QWA, QWB |
| Surgical site infection, deep | |  | JWC, QWC, JAJ, TJA40 |
| Bleeding, superficial or deep | |  | JWD, JWE, QWD, QWE |
| Stoma formation | |  | JFF |
| Reconstruction of urinary tract | |  | KBH, KCH |
| Revision of stoma | |  | JFG4-9 |
| Upper gastrointestinal ulceration (bleeding or perforation) | |  | JDA: 32, 35, 45, 60, 61  JDH: 15, 18, 25, 70, 71 |
| Other reoperation (lavage, drainage, diagnostics, urinary reconstruction etc.) | |  | JAH, JAK, JWW, TJA20, JAD, JAL1,  JFB, JFC, JFH, JFK, JFL, JGA6, KGB, JGC  JFA: 58, 63, 68, 7, 8, 9 |

**Supplementary table 3**: Hazard ratio (HR) for reoperation and mortality following elective colonic resection or stoma formation due to chronic or recurrent diverticular disease.

*Adjusted HRs: estimates for sex, age group, CCI score, severity (elective surgeries only), disease duration, year of surgery, approach, and surgical procedure are mutually adjusted. HRs for the number of contacts and admission count were calculated separately and adjusted for the abovementioned variables, excluding disease duration.

a) Complicated if any contact before or at the resection was with abscess, perforation, stenosis, or fistula

b) 2016-2021 includes 5 years and 9 months, whereas the other 4 groups include 5 years only

| HR (95% CI) | | **Reoperation** | | **Mortality** | |
| --- | --- | --- | --- | --- | --- |
|  |  | Crude HR | Adjusted* HR | Crude HR | Adjusted* HR |
| **Sex** | Female | — | — | — | — |
|  | Male | 1.35 (1.16-1.58) | 1.32 (1.12-1.55) | 0.94 (0.57-1.55) | 1.40 (0.83-2.38) |
| **Age group** | < 60 | — | — | — | — |
|  | 60-69 | 0.94 (0.78-1.13) | 0.98 (0.81-1.19) | 3.72 (1.46-9.52) | 3.70 (1.44-9.54) |
|  | 70-79 | 1.02 (0.83-1.24) | 1.03 (0.84-1.27) | 6.48 (2.62-16.1) | 5.47 (2.16-13.8) |
|  | 80+ | 1.01 (0.73-1.40) | 1.00 (0.71-1.40) | 25.3 (10.3-62.4) | 17.7 (6.88-45.5) |
| **CCI score** | 0 | — | — | — | — |
|  | 1-2 | 0.98 (0.82-1.15) | 0.97 (0.82-1.15) | 0.97 (0.58-1.63) | 0.89 (0.53-1.51) |
|  | 3+ | 1.03 (0.78-1.36) | 1.03 (0.78-1.37) | 0.51 (0.16-1.64) | 0.53 (0.16-1.71) |
| **Severity** | Uncomplicated | — | — | — | — |
|  | Complicated ^a^ | 1.16 (1.0-1.36) | 1.06 (0.90-1.25) | 1.92 (1.18-3.15) | 1.37 (0.82-2.28) |
| **Disease duration** (time from first relevant hospital contact to surgery) | 0-30 days | — | — | — | — |
|  | 30-364 days | 1.01 (0.79-1.29) | 1.08 (0.84-1.39) | 0.40 (0.21-0.75) | 0.53 (0.28-1.00) |
|  | 1+ years | 0.91 (0.71-1.17) | 1.06 (0.82-1.37) | 0.43 (0.23-0.80) | 0.59 (0.31-1.13) |
| **Contact count** (number of relevant hospital contacts before surgery) | 1 | — | — | — | — |
|  | 2 | 1.11 (0.87-1.43) | 1.19 (0.93-1.53) | 0.48 (0.23-1.00) | 0.61 (0.29-1.27) |
|  | 3 | 1.02 (0.79-1.33) | 1.18 (0.90-1.55) | 0.57 (0.27-1.17) | 0.89 (0.42-1.89) |
|  | 4+ | 1.06 (0.86-1.32) | 1.28 (1.02-1.61) | 0.44 (0.24-0.79) | 0.64 (0.34-1.20) |
| **Admission count** (number of admissions due to diverticular disease before surgery) | 0 | — | — | — | — |
|  | 1 | 0.97 (0.79-1.18) | 0.94 (0.77-1.16) | 1.16 (0.63-2.15) | 1.00 (0.53-1.87) |
|  | 2 | 0.78 (0.61-1.00) | 0.81 (0.63-1.03) | 0.87 (0.41-1.85) | 0.92 (0.43-1.99) |
|  | 3+ | 1.15 (0.93-1.43) | 1.24 (1.00-1.55) | 0.87 (0.42-1.82) | 1.02 (0.48-2.16) |
| **Year of surgery** | 1996-2000 | — | — | — | — |
|  | 2001-2005 | 1.11 (0.85-1.44) | 1.13 (0.87-1.48) | 0.66 (0.31-1.39) | 0.82 (0.39-1.72) |
|  | 2006-2010 | 0.98 (0.75-1.27) | 1.09 (0.83-1.43) | 0.45 (0.20-0.99) | 0.55 (0.25-1.25) |
|  | 2011-2015 | 0.98 (0.75-1.27) | 1.13 (0.86-1.50) | 0.77 (0.39-1.52) | 1.07 (0.51-2.24) |
|  | 2016-2021 ^b^ | 0.69 (0.53-0.89) | 0.84 (0.63-1.12) | 0.27 (0.12-0.62) | 0.40 (0.17-0.97) |
| **Approach** | Minimally invasive | — | — | — | — |
|  | Open | 1.60 (1.35-1.90) | 1.48 (1.21-1.80) | 3.51 (1.79-6.89) | 2.63 (1.25-5.54) |
| **Surgical procedure** | Resection, no stoma | — | — | — | — |
|  | Resection with stoma | 1.28 (1.04-1.57) | 1.16 (0.94-1.43) | 3.48 (2.02-6.00) | 2.09 (1.20-3.66) |
|  | Stoma, no resection | 1.43 (0.96-2.14) | 1.49 (0.99-2.25) | 8.52 (4.21-17.3) | 4.81 (2.28-10.2) |
